# Pharmacological properties of ginger (*Zingiber officinale*): what do meta-analyses say? a systematic review

**DOI:** 10.3389/fphar.2025.1619655

**Published:** 2025-07-30

**Authors:** Keshab Raj Paudel, Jake Orent, Olivia Grace Penela

**Affiliations:** ^1^ Department of Biomedical Sciences, Burrell College of Osteopathic Medicine, Melbourne, FL, United States; ^2^ Department of Biomedical Sciences, Mercer University School of Medicine, Columbus, GA, United States

**Keywords:** ginger, gingerol, inflammatory, oxidative stress, pharmacological property

## Abstract

This review evaluates findings from meta-analyses on the pharmacological effects of ginger (*Zingiber officinale*), focusing on inflammation, type 2 diabetes mellitus (T2DM), oxidative stress, and pregnancy-associated nausea and vomiting (NVP). A systematic PubMed search identified relevant meta-analyses published between 2010 and 2025. Ginger supplementation was associated with significant reductions in circulating C-reactive protein (CRP), high-sensitivity CRP, and tumor necrosis factor-α, indicating anti-inflammatory activity. In patients with T2DM, ginger significantly lowered glycosylated hemoglobin 1c and fasting blood glucose. Furthermore, antioxidant effects were observed through reduced malondialdehyde levels and increased glutathione peroxidase activity. In pregnant women, ginger significantly alleviated nausea symptoms but had no significant effect on vomiting frequency. Doses of 1–3 g daily were commonly used for anti-inflammatory, antioxidant, and antidiabetic purposes, while 500–1,500 mg daily in divided doses was used for NVP. Belching was identified as a potential side effect in pregnant women. Despite encouraging outcomes, high heterogeneity in one metanalysis (I^2^ = 98.1%) and potential risk of bias in ‘blinding’ and ‘other bias’ categories across studies reported by another metanalysis highlight the need for further high-quality large-scale randomized controlled trials and meta-analyses to confirm the clinical benefits of ginger.

## 1 Introduction

Ginger (*Zingiber officinale*) has been widely studied for its potential therapeutic effects in various health conditions. This review summarizes evidence from meta-analyses examining its role in reducing inflammation, improving glycemic control in type 2 diabetes, combating oxidative stress, and managing pregnancy-associated nausea and vomiting.

Humans have long recognized the utility of herbs and spices, using them to prevent food spoilage, improve culinary experiences, and promote health (Unuofin et al., 2021; Alipour et al., 2017). In recent years, their popularity has grown based on the belief that they offer effective alternatives to synthetic pharmaceuticals and are associated with fewer adverse effects (Unuofin et al., 2018; Masuku et al., 2020; Unuofin and Lebelo, 2021; Shahrajabian et al., 2019; Singletary, 2010; Kate and Sutar, 2020). Ginger’s pharmacological effects are due to its bioactive compounds, including 6-gingerol, gingerdiol, gingerdione, paradols, shogaols, sesquiterpenes, and zingerone, as well as various phenolics and flavonoids (Unuofin et al., 2021). These constituents are reported to have anti-inflammatory, antioxidant, hypoglycemic and anti-emetic properties (Unuofin et al., 2021; Schepici et al., 2021; Wang et al., 2017; Gaur et al., 2022).

Cardiovascular disease (CVD), a global health concern (Khan et al., 2018), is closely associated with elevated inflammatory markers such as interleukin-6 (IL-6), tumor necrosis factor-alpha (TNF-α), and C-reactive protein (CRP) (Lorenzatti and Servato, 2019). Increased levels of these markers come from increased expression of immune system factors, including nuclear factor-kappa-B (NF-κB) and peroxisome proliferator-activated receptor-gamma (PPAR-γ) (Aboonabi et al., 2020). Proinflammatory cytokines may also increase serum levels of adhesion molecules, which can contribute to peritoneal membrane fibrosis and angiogenesis (Tohma et al., 2017; Pourmasoumi et al., 2018). Vascular cell adhesion molecule-1 (VCAM-1) and intercellular adhesion molecule-1 (ICAM-1) can serve as predictors for CVD (Rahimlou et al., 2019). High sensitivity C-reactive protein (hs-CRP) is involved in the development of insulin resistance, diabetes mellitus, and metabolic syndrome, underlining the importance of suppressing inflammation in the management of chronic diseases (de Lima et al., 2018; Mozaffari-Khosravi et al., 2016). Studies show that ginger can lower fasting blood glucose and improve blood lipids by enhancing antioxidant enzyme function (Chaitanya et al., 2010) as it contains over 40 antioxidants (Firuzi et al., 2011). Clinical trials have demonstrated ginger’s ability to reduce oxidative stress and inflammation by inhibiting NF-κB translocation (Romeo et al., 2004; Krishnamurthy and Wadhwani, 2012; Finkel and Holbrook, 2000).

Oxidative stress plays a central role in aging and many age-related diseases including dementia, arthritis, osteoporosis, and cancer, which can result in the reduced quality of life and increased economic burden on societies (Li and Shah, 2004). Oxidative stress refers to an imbalance between the production of free radicals and the body’s antioxidant capacity (Pounis et al., 2013; Ali et al., 2008). It occurs when the net amount of reactive oxygen species (ROS) and reactive nitrogen species (RNS), which are by-products of normal body function and metabolism, exceeds the intrinsic antioxidant enzymes—superoxide dismutase, catalase, and glutathione peroxidase (GPx)—or extrinsic antioxidants supplied by food (Thamlikitkul et al., 2017).

Oxidative stress is a deleterious process that can occur following an increased level of ROS, RNS, impaired enzyme activity, or lower intake of extrinsic antioxidants, and can mediate damage to critical biological macromolecules like membrane lipids, proteins, and nucleic acids (Butt and Sultan, 2011). Therefore, assigning an approach to reverse such conditions in the body is of great importance for the prevention of consequent diseases. Consumption of fruits and vegetables rich in antioxidants and phytochemical compounds has been associated with reduced concentrations of oxidative stress biomarkers (Stoner, 2013).

Although previous studies have examined the individual effects of ginger-derived bioactive compounds on oxidative stress, the potential synergistic interactions within whole ginger preparations warrant further attention (Duthie and Crozier, 2000). A wide range of biological activities has been demonstrated for ginger consumption including anti-inflammatory, antifungal, antibacterial, antiemetic, anti-cancer, neuroprotective, anti-obesity effects, as well as respiratory system protection (Ho et al., 2013; Akinyemi et al., 2015; Suk et al., 2017; Wei et al., 2017; Zhang et al., 2016). In addition, previous studies and Food and Drug Administration reports have suggested that commonly used dosages of ginger are safe (Tian and Hong, 2012).

The results regarding the effect of ginger supplementation on oxidative stress are inconsistent. While several clinical trials have shown that ginger supplementation can significantly decrease oxidative stress biomarkers (Atashak et al., 2014; Shidfar et al., 2015), others did not provide any significant effect (Attari et al., 2015; Mashhadi et al., 2013). A most recent meta-analysis showed that ginger supplementation has a significant effect on oxidative stress markers (Jalali et al., 2020).

With the changes in lifestyle and living environment, type 2 diabetes mellitus prevalence is increasing at an alarming rate. As indicated by the data given by the International Diabetes Federation, the number of diabetic patients had increased to 415 million by 2015 and in 2040 they projected that 10% of adults will suffer from diabetes (International Diabetes Federation, 2017). Meanwhile, metabolic syndrome (MetS), an important risk factor for diabetes, increased approximately fivefold the risk of type 2 diabetes mellitus (T2DM), and its incidence is as high as one-quarter of the world’s adult population (International Diabetes Federation, 2017). These conditions pose a serious public health threat (Hui et al., 2010). Patients with T2DM or MetS share common characteristics of elevated blood glucose, decreased insulin sensitivity, obesity, dyslipidemia, and hypertension, which often appear simultaneously rather than alone (Grundy et al., 2005).

Wang et al., 2017 indicated that ginger was a promising therapy for T2DM and MetS through multiple targets and pathways. This positive effect may result from its primary bioactive ingredients such as gingerols, shogaols, zingerone, and paradols (Butt and Sultan, 2011). However, findings have been inconsistent; for example, Bordia et al. (1997) reported no significant changes in blood glucose or lipid levels in patients with coronary artery disease after taking 4 g of ginger powder for 3 months (Rahimlou et al., 2019).

Pregnancy-associated nausea and vomiting (NVP) is commonly referred to as morning sickness (although it can occur at any time of the day or night) and affects about 80%–90% of pregnant women in varying degrees (Ebrahimi et al., 2010; Badell et al., 2006). Morning sickness presents in different ways: most women experience both nausea and vomiting, some only nausea without vomiting or retching, but vomiting alone is rare (Badell et al., 2006). Symptoms usually appear at 4–9 weeks of gestation, reaching a peak at 7–12 weeks, and usually subsiding by week 16. About 15%–30% of pregnant women’s symptoms will persist beyond 20 weeks, or even up to the time of delivery, which is then called hyperemesis gravidarum (HG) (Ebrahimi et al., 2010; Badell et al., 2006). HG is severe and persistent vomiting during pregnancy, which can lead to dehydration, electrolyte disturbances, liver damage, possible fetal damage, and in extreme cases, the death of the mother (Ebrahimi et al., 2010; Fagen, 2002). Women with HG usually need to be hospitalized (Ebrahimi et al., 2010), and it occurs in approximately 2% of pregnancies (Ebrahimi et al., 2010; Badell et al., 2006).

Pharmacological treatment of NVP is complicated because during pregnancy, many physiological changes occur, including gastrointestinal motility, plasma volume, and glomerular filtration (Parboosingh, 1981). These factors all influence the distribution, absorption, and excretion of drugs, and due to this reason, not all drugs are safe during pregnancy. Many drugs cross the placenta by simple diffusion and can affect the fetus directly (Parboosingh, 1981). Non-drug therapy of NVP includes ginger and simple lifestyle changes that have been described in the literature (Ebrahimi et al., 2010), besides treatment with vitamin B6 and H1-receptor antagonists (e.g., doxylamine) (Conover, 2002).

So, Ginger (*Zingiber officinale*) has been traditionally used for its diverse therapeutic properties, and modern research suggests its bioactive compounds possess anti-inflammatory, antioxidant, hypoglycemic, and anti-emetic effects. These mechanisms are relevant to major health issues such as type 2 diabetes, inflammatory and oxidative stress-related conditions, and pregnancy-associated nausea and vomiting (NVP) (Krishnamurthy and Wadhwani, 2012; Stoner, 2013; Thamlikitkul et al., 2017; Wang et al., 2017), all of which pose significant clinical and public health challenges. Investigating ginger’s role in these conditions may help validate its clinical utility and support its integration into evidence-based complementary medicine.

This review synthesizes evidence from meta-analyses published between 2010 and 2025, offering a high level of insight into the pharmacological effects of ginger. By pooling data from multiple studies, it enhances statistical power and improves the generalizability of findings. The structured analysis highlights key efficacy and safety outcomes while assessing heterogeneity and study quality across inflammation, T2DM, oxidative stress, and NVP.

The findings of this review have significant translational potential across multiple industries. Ginger’s bioactive compounds may be developed into therapeutic agents for managing chronic inflammatory, metabolic, and gastrointestinal conditions, and can also be incorporated into functional foods and nutraceuticals to promote metabolic health. As demand grows for natural, plant-based remedies with fewer side effects, evidence-based validation of ginger’s efficacy will support its broader adoption in pharmaceuticals, nutrition, and wellness products.

Therefore, the present study aims to synthesize findings from recent meta-analyses on the effects of ginger on inflammatory markers, type 2 diabetes mellitus, oxidative stress, and NVP.

## 2 Methods

**FIGURE 1:**
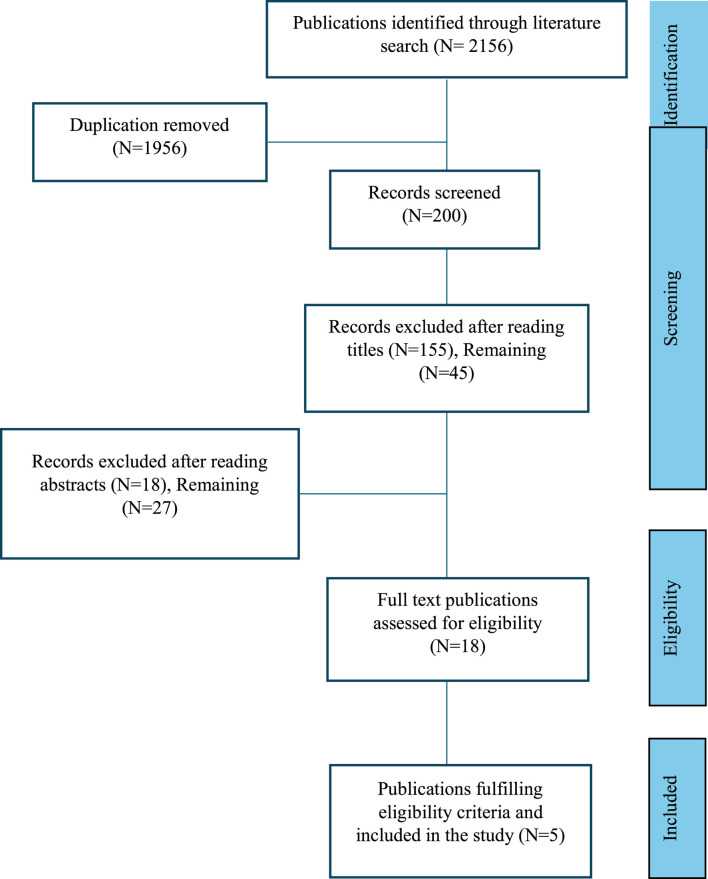
Steps in identification, screening, and inclusion of the published articles (meta-analyses), PRISMA flow chart.

A comprehensive literature search was conducted in PubMed, EMBASE, Scopus, ISI Web of Science, and Cochrane for meta-analyses published between 1 January 2010, and 31 March 2025. The search focused on the effects of ginger (*Zingiber officinale*) on inflammatory markers, type 2 diabetes mellitus (T2DM), oxidative stress, and NVP. Keywords used included “ginger,” “Zingiber officinale,” “phytochemicals,” “bioactive,” “clinical,” “gingerols,” and “shogaols.” The terms “phytochemicals,” “bioactive,” and “clinical” were individually combined with both “ginger” and “Zingiber officinale.” The process of identification, screening, and inclusion of eligible meta-analyses is outlined in Figure 1. Studies were included if they were: (1) published in English; (2) meta-analyses; and (3) evaluated the effects of ginger on inflammatory markers, T2DM and metabolic syndrome, oxidative stress, or NVP. An initial electronic database search yielded a total of 2,156 results. After duplicates were removed, a total of 1956 studies were excluded. After title evaluation, 155 studies were excluded and after reading abstracts 18 studies were excluded. Eighteen full-text articles were examined and five studies fulfilling eligibility criteria were included in the study, and The Preferred Reporting Items for Systematic Reviews and Meta-Analyses (PRISMA) flow chart for this systematic review is shown in Figure 1.

## 3 Results

Table 1 shows the list of meta-analyses, number of corresponding RCTs, sample sizes, dosing regimens, duration of ginger therapy, and observed effects and parameters in the respective meta-analyses. Morvaridzadeh et al. (2020) demonstrated that the ginger had significant impact to lower circulating CRP, hs-CRP and TNF-α levels. Zhu et al. (2018) showed that the ginger caused significant reduction in glycosylated hemoglobin (HbA1c) and fasting blood glucose (FBG). Likewise, findings from Sheikhhossein et al. (2021) illustrated that there was significant reduction in malondialdehyde (MDA), and significant increase in glutathione peroxidase (GPx) with ginger use. Additionally, Viljoen et al. (2014) and Gaur et al. (2022) showed the ameliorating effects of ginger in NVP.

**TABLE 1:** Meta-analyses on effects of ginger on inflammatory markers, type 2 diabetes mellitus and components of the metabolic syndrome, oxidative stress and pregnancy-associated nausea and vomiting.

Meta-analysis	Effects	Parameters
Morvaridzadeh et al. (2020) No. of RCTs: 16 No. of Patients: 1,010 Dose of ginger (gm): 1–3 Duration: 4–12 weeks	Significant impact of ginger in lowering circulating CRP, hs-CRP and TNF-α levels	Significant reduction in C-reactive protein (MD = −5.11, 95% CI = −7.91 to −2.30, P < 0.05, I^2^ = 98.1%), high sensitivity C-reactive protein (MD = −0.88, 95% CI = −1.63 to −0.12, P < 0.05, I^2^ = 90.8%) and TNF-α levels (MD = −0.85, 95% CI = −1.48 to −0.21, P < 0.05, I^2^ = 89.4%), and non-significant effect on IL-6 (MD = −0.45, 95% CI = −1.29–0.38, P > 0.05, I^2^ = 89.2%), soluble intercellular adhesion molecule levels (MD = −0.05, 95% CI = −0.36–0.26, P > 0.05, I^2^ = 00.0%)
Zhu et al. (2018) No. of RCTs: 10 No. of Patients: 490 Dose of ginger (gm): 1–3/d Duration: 30 days- 3 months	Significant reduction in glycosylated hemoglobin (HbA1c), significant reduction in fasting blood glucose (FBG)	Significant reduction in glycosylated hemoglobin (HbA1c) (MD = −1.00, 95% CI = −1.56 to −0.44, P < 0.001)); significant reduction in FBG in T2DM patients (MD = −21.24, 95% CI = −33.21 to −9.26, P < 0.001)
Sheikhhossein et al. (2021) No. of RCTs: 12 No. of Patients: 542 Dose of ginger (gm): 0.5–3/d Duration: 6–12 weeks	Significant reduction in malondialdehyde (MDA) and significant increase in glutathione peroxidase (GPx), and no significant alteration in total antioxidant capacity (TAC)	Significant reduction in MDA (MD = 1.45, 95% CI = 2.31–0.59, P = 0.001), significant increase in GPx (MD = 1.93, 95% CI = 0.20–3.66, P = 0.029), no significant increase in TAC (MD = 0.42, 95% CI = 0.03–0.88, P = 0.069)
Viljoen et al. (2014) No. of RCTs: 12 No. of Patients: 1,278 pregnant females Dose of ginger (gm): 0.125–1.05 single or up to 4 times a day Duration: 4–21 days	Potential benefits of ginger in reducing nausea symptoms in pregnancy, ginger did not significantly affect vomiting episodes	Ginger significantly improved the symptoms of nausea when compared to placebo (MD = 1.20, 95% CI = 0.56–1.84, P = 0.0002, I^2^ = 0%). Ginger did not significantly reduce the number of vomiting episodes during NVP, when compared to placebo, although there was a trend towards improvement (MD = 0.72, 95% CI = −0.03–1.46, P = 0.06, I^2^ = 71%)
Gaur et al. (2022) No. of RCTs: 7 No. of Patients: 819 Dose of ginger (gm): 1–1.5/d Duration: 4 days-3 weeks	Ginger can be used to manage NVP as same amount as vitamin B6	Ginger has no notable influence on the intensity of nausea scores (MD = −0.15, 95% CI = −0.35–0.05, P = 0.14, I^2^ = 50.0%), reducing vomiting scores (MD = 0.05, 95% CI = −0.11–0.21, P = 0.57, I^2^ = 0%) compared with vitamin B6, however, when compared to ginger, vitamin B6 therapy had a significant influence on improving total NVP results (MD = 0.36, 95% CI = 0.06–0.65, P = 0.02, I^2^ = 17%)

MD, mean difference; CI, confidence interval; RCTs, randomized controlled trials; I^2^, heterogenicity of RCTs; GPx, glutathione peroxidase; NVP, nausea vomiting during pregnancy; CRP, C-reactive protein; hs-CRP, high sensitivity C-reactive protein; TNF-α, tumor necrosis factor-alpha; MDA, malondialdehyde; TAC, total antioxidant capacity; FBG, fasting blood glucose; HbA1c, glycosylated hemoglobin.

Table 2 illustrates the dose duration of ginger therapy, and age and BMI of the participants in various RCTs evaluating its effects on inflammatory markers, as reported in the meta-analysis by Morvaridzadeh et al. (2020). Patients were generally overweight or obese, with mean ages ranging from 31.62 ± 6.0 to 57.98 ± 6.2 years (Table 2). Table 3 summarizes the dose, duration of ginger supplementation, and participant characteristics in RCTs assessing its effects on T2DM and metabolic syndrome, as reported by Zhu et al. (2018). The dose of ginger was 1–3 g; age was 20–80 years, and BMI was 20–40 (Table 3).

**TABLE 2:** Dose, duration of ginger therapy, and age and BMI of the participants in various RCTs to observe effects of ginger on inflammatory markers (a meta-analysis by Morvaridzadeh et al., 2020).

Dose of ginger (gm)	Duration (weeks)	Age (mean ± SD)	BMI
1	12	52.6 ± 8.4	26.8
1.6	6	46.4 ± 5.5	32.77
2	8	55.21 ± 1.1	28.4
3	8	55.21 ± 1.1	28.4
3	6	56.00 ± 2.5	27
3	10	57.98 ± 6.2	27.18
3	8	52.81 ± 6.44	29.8
1	4	31.62 ± 6.0	25.5
2	8	49.27 ± 5.18	25.5
3	8	57.98 ± 6.2	30.94
2	12	50.04 ± 10.2	31.53
1	12	45.45 ± 2.31	29.2
1.5	12	45.2 ± 7.64	27
2	12	56.00 ± 2.5	29.23
2	10	51.74 ± 8.58	27

RCTs, randomized controlled trials; BMI, body mass index; SD, standard deviation.

**TABLE 3:** Dose, duration of ginger therapy, and age and BMI of the patients in various RCTs conducted to observe effects of ginger on type 2 diabetes mellitus and metabolic syndrome (a meta-analysis by Zhu et al., 2018).

Dose of ginger (gm)	Duration (days)	Age (range)	BMI (kg/m^2^)
3	45	20–80	NA
3	30	40–60	NA
1.6	84	30–70	20–35
2	60	38–65	29.5
3	56	30–70	<40
3	90	20–60	≤30
1	70	18–30	≥30
2	84	18–45	30–40
2	84	18–45	29.78 ± 3
3	42	30–60	NA
1	70	29–79	NA
1	70	29–79	NA

RCTs, randomized controlled trials; BMI, body mass index; NA, not available.

Table 4 displays dose and duration of ginger therapy, and participant demographics for RCTs examining effects of ginger on biomarkers of oxidative stress in the meta-analysis conducted by Sheikhhossein et al. (2021).

**TABLE 4:** Dose and duration of ginger therapy, and age and BMI of patients in various RCTs to observe effects of ginger on biomarkers of oxidative stress (a meta-analysis by Sheikhhossein et al., 2021).

Dose of ginger (gm)	Duration (weeks)	Age (mean)	BMI (kg/m2)
3	6	19	25
3	6	48	30
1	10	24	25
2	10	24	25
3	12	40	27
2	12	31	25
1	10	57	26
3	12	36	26
0.5	9	52	35
2	12	40	35
2	8	45	35
1.5	12	49	31

RCTs: randomized controlled trials, BMI: body mass index.

Similarly, Table 5 outlines dose, frequency, and duration of ginger therapy in the treatment of pregnancy-associated nausea and vomiting as reported in the meta-analysis by Viljoen et al. (2014).

**TABLE 5:** Dose and frequency, and duration of ginger therapy in the treatment of pregnancy-associated nausea and vomiting (Viljoen et al., 2014).

Dose and frequency	Duration (days)
500 mg 5 times a day	4
350 mg 3 times a day	4
500 mg 2 times a day	4
250 mg 4 times a day	4
250 mg 4 times a day	14
200 mg 3 times a day	5
250 mg 4 times a day	4
500 mg 2 times a day	7
300 mg 3 times a day	21
500 mg 3 times a day	3
250 mg 4 times day	4
125 mg 4 times a day	4

Table 6 summarizes the adverse events reported in pregnancy-related RCTs comparing ginger, placebo, and vitamin B6. Notably, belching was the only side effect significantly associated with ginger (Viljoen et al., 2014).

**TABLE 6:** Adverse events and side-effects of ginger versus control group (Placebo, Vitamin B6) (Viljoen et al., 2014).

Ginger versus placebo	Ginger versus vitamin B6
Allergic reaction (Willetts et al., 2003)^#^	Arrhythmia (Chittumma et al., 2007)^#^
Dehydration (Willetts et al., 2003)^#^	Spontaneous abortions (Ensiyeh and Sakineh, 2005; Smith et al., 2004)^#^
Spontaneous abortions (Vutyavanich et al., 2001; Willetts et al., 2003)^#^	Belching (Smith et al., 2004)*
Abdominal discomfort (Vutyavanich et al., 2001)	Burning sensation after capsule ingestion (Smith et al., 2004)
Diarrhea (Vutyavanich et al., 2001)	Drowsiness (Chittumma et al., 2007; Sripramote and Lekhyananda, 2003)
Drowsiness (Vutyavanich et al., 2001)	Dry retching (Smith et al., 2004)
Headache (Vutyavanich et al., 2001)	Heartburn (Chittumma et al., 2007; Sripramote and Lekhyananda, 2003)
Heartburn (Vutyavanich et al., 2001; Basirat et al., 2009; Willetts KE et al., 2003)	Vomiting (Smith et al., 2004)
Worsening symptoms requiring pharmaceutical treatment (Willetts et al., 2003)	

#Major adverse events (serious complications, possibly detrimental to the mother or fetus) (authors’ judgement); rest considered minor (discomfort, but manageable side effects) - sorted alphabetically, first major then minor events (Viljoen et al., 2014).

*Indicates statistically significant finding: Ginger significantly increased the risk of belching compared to vitamin B_6_ (Viljoen et al., 2014).

Table 7 presents ginger dosing, frequency, and duration of therapy used in RCTs evaluating anti-emetic effects in pregnancy from the meta-analysis by Gaur et al. (2022). Dosing regimens, treatment durations, and participant demographics were comparable across the five meta-analyses, except for those focused on NVP (Table 7).

**TABLE 7:** Dose, frequency and duration of ginger therapy in various RCTs to observe anti-emetic effects of ginger in nausea and vomiting of pregnancy (Gaur et al., 2022).

Dose and frequency (gm/day)	Duration (days)
1	4
1	4
1	4
1	4
1.95	4
1.05	21
1.5	21

## 4 Discussion

This study aimed to synthesize findings from meta-analyses on effects of ginger on inflammatory markers, type 2 diabetes mellitus, oxidative stress, and pregnancy-associated nausea and vomiting. Morvaridzadeh et al. (2020) reported a significant reduction of circulating C-reactive protein (CRP), high-sensitivity C-reactive protein (hs-CRP), and tumor necrosis factor alpha (TNF-α), with high heterogeneity scores (I^2^ = 98.1%, 90.8%, and 89.4% respectively), suggesting substantial variability among the results of included RCTs. Additionally, the effect of ginger supplementation was not associated with a change in IL-6 and soluble intercellular adhesion molecule levels (Table 1). A previous study has shown similar effects on CRP and TNF-α levels but differed in their findings on IL-6 levels (Jalali et al., 2020). These discrepancies may be due to the variation in chemical composition of the ginger products, which can vary by preparation method, cultivation region, and storage conditions (Jolad et al., 2005). Additionally, it can be hypothesized that interpersonal pharmacokinetic and pharmacodynamic variations among the individuals based on their genetic make-up might have continued to the variation in the responses.

Zhu et al. (2018) reported significant reductions in glycosylated hemoglobin (HbA1c) and fasting blood glucose (FBG) among T2DM patients, with low inter-study heterogeneity. These findings were consistent with prior studies in diabetic animal models showing improved glucose tolerance and decreased FBG following ginger administration (Nammi et al., 2009; Madkor et al., 2011; Islam and Choi, 2008). The underlying mechanism involved the inhibition of α-glucosidase and α-amylase, key enzymes in complex carbohydrate digestion and absorption (Priya Rani et al., 2011). Besides, 6-Gingerol, a component of ginger, showed potent activity in stimulating glucose metabolism via the AMPK alpha2-mediated AS160-Rab5 pathway and through potentiation of insulin-mediated glucose regulation (Lee et al., 2015). 6-Paradol and 6-shogaol, which are the pungent compounds of ginger, also have the same effects (Wei et al., 2017). Ginger extraction could also enhance the expression of glucose transporter type 4 (GLUT-4) (Rani et al., 2012) and increase GLUT-4 to promote glucose uptake in adipocytes and skeletal muscle cells (Li et al., 2012). Insulin sensitivity could be enhanced by upregulating adiponectin and peroxisome proliferative activated receptor γ (Isa et al., 2008), and by ginger constituent interaction with the 5-HT3 receptor (Heimes et al., 2009). Moreover, 6-Gingerol extracted from ginger could protect pancreatic β-cells (Chakraborty et al., 2012). The reduction in HbA1c suggests a potential long-term hypoglycemic effect of ginger (Zhu et al., 2018). So, the ginger appears to have hypoglycemic effects via various mechanisms-mainly GLUT4, adiponectin and peroxisome proliferative activated receptor γ and pancreatic β-cells.

The meta-analysis by Sheikhhossein et al. (2021) found that there was significant reduction in malondialdehyde (MDA) and significant increase in GPx, and no significant alteration in total antioxidant capacity (TAC). This finding on MDA level is consistent with previous findings by Jalali et al. (2020), but the TAC finding is not in agreement with that reported by the same study. Again, this discrepancy in findings might be in part due to variation in the chemicals of ginger products and other pharmaceutical factors (Jolad et al., 2005). However, future studies are recommended to explore the reason for this discrepancy.

Ginger extract demonstrated antioxidant effects in human chondrocyte cells under interleukin-1β-mediated oxidative stress. It upregulated the expression of several antioxidant enzymes and reduced the generation of ROS and lipid peroxidation (Hosseinzadeh et al., 2017). Additionally, the ginger extract could decrease the production of ROS in human fibrosarcoma cells with H_2_O_2_-induced oxidative stress (Romero et al., 2018). In stressed rat heart homogenates, ginger strain decreased the content of MDA, which was related to lipid peroxidation (Akinyemi et al., 2013). Ginger and its bioactive compounds (such as 6-shogaol) exhibited antioxidant activity via the nuclear factor erythroid 2-related factor 2 (Nrf2) signaling pathway (Fathi et al., 2021). The potential mechanism for 6-shogaol function is that it can lead to the translocation of Nrf2 into the essence and enhance the expression of Nrf2 target genes by modifying Keap1 (Kelch-like ECH-associated protein 1), a cytoplasmic regulatory protein that plays a central role in controlling the Nrf2 pathway, which is essential for the cellular response to oxidative stress. Thus, the level of reduced glutathione increases, and the level of ROS decreases. Additionally, besides antioxidant activity via the Nrf2 pathway, ginger supplementation increases the antioxidant defense activity by activating Nuclear Factor 2 and increasing the expression of several antioxidant enzymes, thereby decreasing ROS production and lipid peroxidation. Thus, ginger appears to protect against oxidative stress and inhibit the loss of antioxidants (Fathi et al., 2021). So, ginger can prevent the damage to critical biological macromolecules like membrane lipids, proteins, and nucleic acids (Butt and Sultan, 2011) and play a preventive role to decrease the prevalence of diseases resulting from such damage.

Viljoen et al. (2014) showed that ginger significantly improved the symptoms of nausea when compared to placebo (P = 0.0002, I^2^ = 0). However, ginger did not significantly reduce the number of vomiting episodes during NVP when compared to placebo, although there was a trend toward improvement (P = 0.06, I^2^ = 71%). Ginger can increase gastric contractility, speed up gastric emptying, and therefore decrease the gastrointestinal transit time of meals, which can decrease the feeling of nausea (Nikkhah et al., 2018). Likewise, Gaur et al. (2022) reported that ginger has no notable effect on the intensity of nausea scores (I^2^ = 50%) and decreases vomiting scores (I^2^ = 0%) compared with vitamin B6. However, when compared to ginger, vitamin B6 intervention had a significant effect on improving total NVP results (I^2^ = 17%). These symptoms are linked to human chorionic gonadotropin, digestive system functioning, and smell sensitivity during gestation.

The exact cause of NVP remains unclear and is probably multifactorial. Theories include the rapid increase in hormones such as estrogen and human chorionic gonadotropin (Quinlan and Hill, 2003), *Helicobacter pylori* infection, as well as psychological and genetic predisposition (Badell et al., 2006; Quinlan and Hill, 2003). Severe NVP and HG can lead to maternal malnourishment and weight loss, leading to negative fetal outcomes including low birth weight and preterm birth (Ebrahimi et al., 2010). Maternal complications include acute renal failure, esophageal rupture, coagulopathy, and on rare occasions, Wernicke’s encephalopathy (Badell et al., 2006). The negative effects of NVP described clearly show the importance of managing and treating NVP and HG as early as possible and not considering NVP as merely an unpleasant part of pregnancy that must be endured and suffered through.

Although the exact mechanism is not well understood, nutrient deficiencies, particularly vitamin B6, are linked to NVP and can often be corrected through supplementation. Ginger is also widely used as a traditional remedy for NVP globally (Chittumma et al., 2007; Badell et al., 2006). The effectiveness of vitamin B6 in managing overall NVP scores was substantially different from ginger, as one study (Gaur et al., 2022) found vitamin B6 to be more effective than ginger in improving overall NVP symptom scores. Thus, ginger can have some preventive role for nausea and vomiting in NVP, however, it may not treat the vomiting episodes in NVP. However, Viljoen et al. (2014) employed the Cochrane “Risk of Bias” assessment tool to evaluate the methodological quality of the randomized controlled trials (RCTs) included in their meta-analysis. The authors reported a high risk of bias particularly in the domains of blinding and other sources of bias. Overall, all 12 included studies were determined to exhibit either a high or moderate risk of bias.

Ginger significantly increased the risk of belching compared to vitamin B6 (RR = 27.18, 95% CI = 1.63–453.06) in one study (Smith et al., 2004). For all other adverse events and side effects reported in the various studies including arrhythmia (Chittumma et al., 2007), spontaneous abortions (Smith et al., 2004; Willetts et al., 2003), burning sensation after capsule ingestion (Smith et al., 2004), drowsiness (Chittumma et al., 2007; Smith et al., 2004), dry retching (Smith et al., 2004), heartburn (Chittumma et al., 2007; Smith et al., 2004), and vomiting (Smith et al., 2004), there were no significant differences observed between the ginger and vitamin B6-treated groups (Viljoen et al., 2014). However, most adverse events were considered minor, and interpretations varied by study authors (Table 4).

Ginger was typically administered at doses of 1–3 g/day for anti-inflammatory, antioxidant, and antidiabetic effects, and at 500–1,500 mg/day (in divided doses up to 2–5 times daily) for managing NVP symptoms (Morvaridzadeh et al., 2020; Zhu et al., 2018; Sheikhhossein et al., 2021; Viljoen et al., 2014; Gaur et al., 2022).

## 5 Limitations

Variations in ginger dosage, composition, and follow-up durations may have contributed to the high heterogeneity observed in some meta-analyses. Conversely, low heterogeneity in certain findings may be attributed to sub-grouping of samples, small sample sizes, or homogenous study populations. Additionally, the presence of publication or selection bias cannot be ruled out. Variations in participants’ dietary habits and nutritional patterns may also have influenced study outcomes.

## 6 Conclusion

Ginger significantly lowers circulating CRP, hs-CRP, and TNF-α levels, indicating its anti-inflammatory potential. It also significantly reduces HbA1c, and FBG, supporting its beneficial effects in T2DM. Ginger supplementation leads to a significant reduction in MDA and a significant increase in GPx, reflecting its antioxidant activity. Ginger significantly improves the nausea symptoms compared to placebo; however, it does not appear to reduce the frequency of vomiting episodes compared to placebo. Ginger may be considered a generally safe and potentially effective non-pharmacological option for women suffering from NVP. Doses of ginger ranging from 500 mg to 1,500 mg per day up to five times daily, may alleviate NVP symptoms. Daily doses of 1–3 g may provide anti-inflammatory, antioxidant, and antidiabetic benefits with minimal side effects, although belching was a noted adverse event among pregnant women. However, potential selection and publication bias among the included RCTs cannot be ruled out. Further large-scale randomized controlled trials and meta-analyses are needed to confirm the pharmacological effects of ginger and establish its clinical indications.

## Data Availability

The original contributions presented in the study are included in the article/supplementary material, further inquiries can be directed to the corresponding author.
